# Investigating Mpox Strain Dynamics Using Computational and Data-Driven Approaches

**DOI:** 10.3390/v17020154

**Published:** 2025-01-23

**Authors:** Isaiah Oke Idisi, Kayode Oshinubi, Vigbe Benson Sewanu, Mukhtar Muhammed Yahaya, Oluwafemi Samson Olagbami, Helen Olaronke Edogbanya

**Affiliations:** 1 Department of Mathematical Sciences, Federal University of Technology, Akure PMB 704, Ondo, Nigeria; 2 Black in Mathematics Association (BMA), Pretoria 0001-0039, South Africa; 3 Department of Mathematics, Alvan Ikoku Federal University of Education, Owerri 460281, Nigeria; 4 Department of Mathematical Sciences, Stellenbosch University, Stellenbosch 7602, South Africa; 5 International Centre for Applied Mathematical Modelling and Data Analytics, Federal University, Oye-Ekiti 371104, Nigeria; 6 Department of Mathematics, Federal University, Oye-Ekiti 371104, Nigeria; 7 Department of Mathematical Sciences, Federal University Lokoja, Lokoja PMB 1154, Kogi, Nigeria

**Keywords:** Mpox transmission dynamics, mathematical modeling, strain-specific epidemiology, data-driven modeling, scenario simulation, statistical fitting

## Abstract

This study explores Mpox transmission dynamics using a mathematical and data-driven epidemiological model that incorporates two viral strains, Clade I and Clade II. The model includes transmission pathways between humans and mammals and divides the human population into susceptible, exposed, infectious, hospitalized, and recovered groups. Weekly data from the WHO for Spain, Italy, Nigeria, and the DRC from 2022 to 2024 are used for model validation via non-linear least-squares fitting, with model performance assessed by Root Mean Squared Error (RMSE). We conduct time-series analysis to detect trends and anomalies in Mpox cases, with scenario simulations examining strain-specific transmission and the basic reproduction number ($R_{0})$. The mathematical model fit is compared with two statistical model fits to emphasize the importance of developing a model that incorporates Mpox strain. Mathematical analysis confirms the model’s key properties, including positivity, boundedness, and equilibrium stability. Results underscore the importance of strain-specific dynamics and varying infection proportions for $R_{0}$. This study combines mathematical rigor with empirical data to provide valuable insights into Mpox transmission and offers a framework for understanding multi-strain pathogens in diverse populations. Results from the simulation indicate that an increase in the effective contact rate leads to the dominance of the prevalent Mpox Clades in each country. Based on these findings, we recommend the implementation of strategies aimed at reducing the effective contact rate to control the spread of the virus strains.

## 1. Introduction

Mpox, formerly known as monkeypox, is a zoonotic viral disease caused by the monkeypox virus (MPXV), a member of the genus *Orthopoxvirus* in the *Poxviridae* family [1,2]. First identified in 1958 in Copenhagen in Denmark in monkeys kept for research, its first human case was reported in 1970 in the Democratic Republic of Congo (DRC) [1,2,3]. Due to concerns over stigmatization, the World Health Organization (WHO) renamed the disease Mpox in 2022 [4]. Endemic to several African countries, including Nigeria and Cameroon, Mpox has two major strains: Clade I (Congo Basin) and Clade II (West African). Clade I is more virulent, with a fatality rate of approximately 10%, while Clade II, linked to recent global outbreaks, is less severe [2,5,6].

Transmission occurs through human-to-human contact, animal-to-human interactions, and environmental exposure to contaminated materials [1,2]. Symptoms include fever, rash, swollen lymph nodes, and fatigue, with an incubation period of 1–21 days [1,2]. Diagnosis primarily relies on polymerase chain reaction (PCR) testing, while vaccination provides preventive benefits [1,2]. Mpox outbreaks impose significant socio-economic burdens, particularly in resource-limited settings, and are often exacerbated by stigma, misinformation, and healthcare system strain [7,8,9]. Effective mitigation strategies include early detection, public education, and understanding the virus’s transmission dynamics [10,11].

Mpox outbreaks have significant socio-economic implications, impacting individuals, healthcare systems, and communities. Affected individuals often face stigma and discrimination, while healthcare systems in resource-limited regions are strained by the demands of managing outbreaks [7,8]. Public anxiety and misinformation further exacerbate these challenges, particularly in non-endemic regions [9]. Effective mitigation strategies include early detection, isolation of cases, public education, and research into the virus’s transmission dynamics [10,11]. Epidemiological research is crucial for developing effective public health responses and intervention strategies. For instance, McCollum et al. [5] presented epidemiological data on Mpox between 2018 and 2021, highlighting the importance of surveillance in understanding disease trends.

Also, the Surveillance Outbreak Response Management and Analysis System (SORMAS) proved invaluable in managing Mpox cases during the 2017-2019 outbreak in Nigeria, aiding data collection and response planning [12]. Furthermore, a case–control study revealed that individuals infected with HIV face increased risk and mortality from Mpox, underscoring the interplay between co-morbid conditions and disease outcomes [13]. Epidemiological investigations in children and adolescents have also highlighted unique clinical and diagnostic challenges for these populations, emphasizing the need for tailored public health strategies [14]. The pathogenesis of Mpox involves complex interactions between the virus and host immune responses. A recent review discussed the biology of Mpox, prevention strategies, and treatment options, providing a comprehensive understanding of the disease [15]. Transmission dynamics, including environmental and zoonotic factors, were further explored, revealing how these contribute to the virus’s spread [16]. Additionally, the occurrence of Mpox in vaccinated individuals or those with prior infections has raised concerns about vaccine efficacy and re-infection risks, as highlighted in recent case reports [17]. The search for effective treatments and preventive measures remains a priority in Mpox research. Investigations into antiviral drugs, such as Atovaquone, Mefloquine, and Molnupiravir, have opened new avenues for therapeutic interventions [18]. A summary of prevention strategies and emerging therapeutic targets highlighted the challenges in vaccine development and treatment implementation [19]. Optimal control theory has also been applied to assess the impact of environmental transmission and propose strategies to reduce infections [20]. Effective public health communication and proactive surveillance systems are crucial for Mpox outbreak containment. An analysis of Nigerian media reporting during the 2017 outbreak revealed gaps in risk communication and public awareness, emphasizing the need for better information dissemination [21].

Mathematical, data-driven, and computational models have played a critical role in understanding Mpox dynamics and forecasting outbreaks, and they have become valuable tools for studying Mpox transmission dynamics and informing control measures. Several studies have provided insights into the epidemiology and dynamics of Mpox outbreaks [3,22,23,24,25,26]. Researchers have used such models to evaluate the effects of behavioral changes, vaccination, and other interventions. For example, in [22,23], the authors modeled the Mpox outbreak among gay, bisexual, and other men who have sex with men (GBMSM), demonstrating that behavioral changes significantly reduced Mpox transmission. A fractional-order SEIR model demonstrated the utility of vaccination in controlling Mpox spread by estimating the basic reproductive number [26]. Hybrid forecasting techniques, combining time-series decomposition and machine learning methods, have been applied to predict infection and death rates with promising results [27]. Moreover, machine learning approaches have been used to estimate underreported cases at the onset of outbreaks, highlighting the role of advanced modeling in public health [28]. A comparative analysis of regression and machine learning models further showcased their respective strengths and limitations in predicting global Mpox trends [29]. In addition, advancements in machine learning and artificial intelligence (AI) provide new opportunities for outbreak prediction, early diagnosis, and targeted prevention strategies. Ferrari et al. [30] developed a bagged ensemble methodology (BaLSTM) for predicting weekly Mpox cases in Brazil, highlighting the potential of AI in infectious disease modeling. Advancements in artificial intelligence have significantly enhanced Mpox diagnostic capabilities. For example, an automated deep learning model using transfer learning has been developed to detect Mpox skin lesions with high accuracy, improving early diagnosis [31]. Several authors have provided valuable reviews that enhance our understanding of disease dynamics. For instance, Chadaga et al. [32] conducted a systematic review emphasizing the potential of AI-based techniques to augment traditional diagnostic workflows. Similarly, Lewis [33] reviewed models addressing tick population dynamics, host biodiversity, Lyme disease risk, spatial tick invasion, and human Lyme disease, while also highlighting the need for research on co-infections with other tick-borne diseases. Banuet-Martinez et al. [11] critically summarized current knowledge from epidemiological mathematical models, within-host models, and between-host transmission models, adopting a One Health perspective. In addition, diagnostic tools such as molecular and immunological assays have been reviewed for their efficacy in real-world settings [34]. Mathematical modeling has also been used to study the peculiar re-emergence of Mpox, stressing the importance of robust surveillance systems and interventions [35,36,37].

Recent outbreaks of different strains of Mpox globally have highlighted the need for comprehensive studies addressing its epidemiology, pathogenesis, modeling, diagnostic techniques, and prevention strategies. The articles reviewed above synthesize recent advancements in Mpox research to provide a multi-disciplinary framework for combating the disease; hence, we propose a computational, mathematical, and data-driven framework to study Mpox strains, which is lacking in the literature. Given the re-emergence of Mpox globally, continued research into its transmission dynamics is essential. This study presents a mathematical model that incorporates multiple transmission pathways and strain-specific dynamics. The model is validated using real-world data from Spain, Italy, Nigeria, and the DRC, contributing to the development of effective public health policies and intervention strategies for Mpox control. The mathematical model fit is compared with two statistical model fits to emphasize the importance of developing a model that incorporates the Mpox strain. By integrating the insights in this study, researchers and policymakers can develop targeted strategies to mitigate the impact of Mpox outbreaks and enhance global preparedness for emerging and re-emerging zoonotic diseases.

## 2. Mathematical Model Formulation

The model we develop in this article takes into consideration the two strains (Clade I and Clade II) of the Mpox virus, which are based on two different infection pathways in the human population of the model. The total human population at time t, denoted by N(t), is subdivided into susceptible humans S(t), exposed humans E(t), infectious humans based on Clade I $I_{1}\left(t\right)$, infectious humans based on Clade II $I_{2}\left(t\right)$, hospitalized humans H(t), and recovered humans R(t). Therefore, the total human population is given by

(1) $$N\left(t\right)=S\left(t\right)+E\left(t\right)+I_{1}\left(t\right)+I_{2}\left(t\right)+H\left(t\right)+R\left(t\right),$$

while the mammal population is subdivided into two compartments, susceptible $S_{m}\left(t\right)$ and infectious $I_{m}\left(t\right)$, such that the total mammal population is given by

(2) $$M\left(t\right)=S_{m}\left(t\right)+I_{m}\left(t\right).$$

The total population of both the human and mammal populations considered in the model dynamics is given as

(3) $$N_{T}\left(t\right)=N\left(t\right)+M\left(t\right).$$

Thus, the transmission model with two strain infection dynamics is represented by the following system of non-linear ordinary differential equations:

(4) $$\begin{array}{cc} \dot{S} & =\Pi_{h}-\left(\lambda_{h}+\mu_{h}\right)S+\epsilon R,\\ \dot{E} & =\lambda_{h}S-\gamma E_{h}-\mu_{h}E_{h}\\ \dot{I_{1}} & =\left(1-\theta \right)\gamma E_{h}-\left(\tau_{1}+\delta_{1}+\mu_{h}\right)I_{1},\\ \dot{I_{2}} & =\theta \gamma E_{h}-\left(\tau_{2}+\delta_{2}+\mu_{h}+\psi \right)I_{2},\\ \dot{H} & =\tau_{1}I_{1}+\tau_{2}I_{2}-\left(\delta_{3}+\mu_{h}+\eta \right)H\\ \dot{R} & =\eta H+\psi I_{2}-\left(\epsilon +\mu_{h}\right)R,\\ \dot{S_{m}} & =\Pi_{m}-\lambda_{m}S_{m}-\mu_{m}S_{m},\\ \dot{I_{m}} & =\lambda_{m}S_{m}-\mu_{m}I_{m}, \end{array}$$

under the initial conditions

(5) $$S\left(0\right)>0,E\left(0\right)\geq 0,I_{1}\left(0\right)\geq 0,I_{2}\left(0\right)\geq 0,H\left(0\right)\geq 0,R\geq 0,S_{m}>0,I_{m}\geq 0,$$

with $\left(S\left(0\right),E\left(0\right)\right),I_{1}\left(0\right),I_{2}\left(0\right),H\left(0\right),R\left(0\right),S_{m}\left(0\right),I_{m}\left(0\right)\in R_{+}^{8}$.

The effective contact rate with an infectious human or infectious mammal is defined as

(6) $$\lambda_{h}=\frac{\beta_{h}\left(I_{1}+\sigma I_{2}\right)}{N_{h}},\lambda_{m}=\frac{\alpha \beta_{hm}\left(I_{1}+\sigma I_{2}\right)}{N_{h}}+\frac{\beta_{m}I_{m}}{N_{m}}.$$

It is pertinent to define the rate of change of cumulative death due to the disease as

$$\dot{D}=\kappa \left|I_{1}\left(t\right)+I_{2}\left(t\right)\right|$$

From which it follows that the weekly mortality in week κ (expressed as $G_{k}$) is given by

(7) $$G_{\kappa }=\int_{\kappa -1}^{\kappa }\dot{D}\left(t\right)\;dt$$

**Figure 1:**
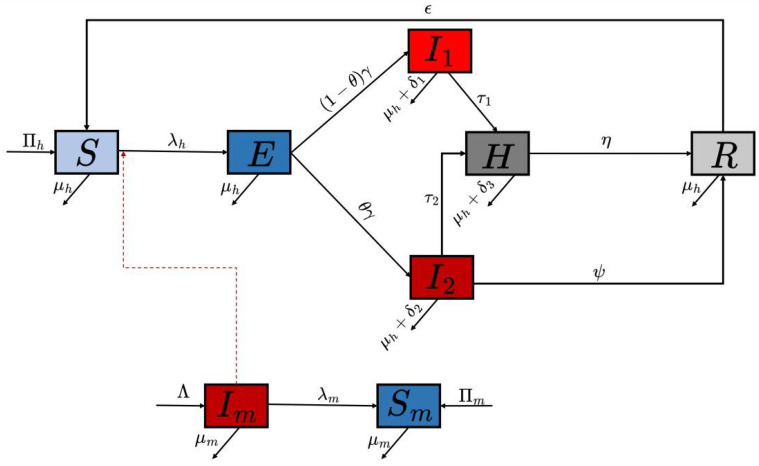
Schematic diagram for dual-strain Mpox model.

The schematic diagram of the model is presented in Figure 1, and the state variables and parameters of the model are described in Table 1.

The population of susceptible humans is generated by immigration at constant rate $\Pi_{h}$ and the inclusion of recovered individuals with loss of immunity at rate ϵ. Natural death occurs in all human classes at a rate of $\mu_{h}$. The populations of exposed humans *E* are generated by the infection of susceptible (*S*) individuals at rate $\lambda_{h}$. This population reduces through progression to the infectious classes $I_{1}$ and $I_{2}$ at rates (1−θ)γ and θγ, respectively, which further decrease due to natural death and disease-induced death at rates $\delta_{1}$ and $\delta_{2}$. The population of infectious humans due to Clade I ($I_{1}$) decreases by hospitalized rate $\tau_{1}$, while the population of infectious humans due to Clade II ($I_{2}$) decreases by hospitalized rate $\tau_{2}$ and recovery rate ψ. Also, the population of hospitalized individuals is generated as a result of individuals’ progression from $I_{1}$ and $I_{2}$, and further decreases due to natural death, disease-induced death $\delta_{3}$, and recovery of individuals at rate η. The recovered human population increases with the recovery of infectious humans from Clade II at a rate of ψ and hospitalization rate η. This population decreases due to natural death and the progression of individuals to susceptible classes at rate ϵ due to immunity loss.

Similarly, the population of susceptible mammals ($S_{m}$) is generated by immigration at rate $\Pi_{m}$ and decreases by infection at rate $\lambda_{m}$, and they die naturally at rate $\mu_{m}$. We assume that the interaction of infected mammals with susceptible mammals leads to infection, and the probability of infection is 1. Finally, the population of infectious mammals $I_{m}$ is generated by the progression of susceptible mammals at rate $\gamma_{m}$. This population is reduced by natural death at the rate $\mu_{m}$.

We assume in our model that there are three pathways of transmission dynamics: human-to-human transmission, mammal-to-human transmission, and mammal-to-mammal transmission.

We present the mathematical analysis of Model (4) in Appendix A.

## 3. Parameter Estimation and Mathematical Model Fitting

**Figure 2:**
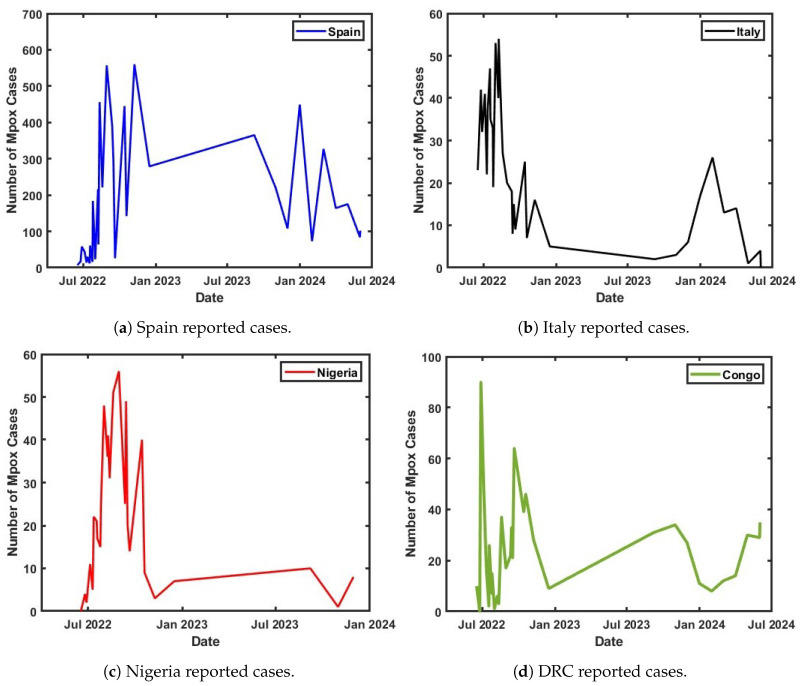
Weekly reported cases of Mpox for Spain, Nigeria, Italy, and the DRC.

The formulated Clades I and II epidemiological model was rigorously validated, with its reliability confirmed through the use of published data from the World Health Organization (WHO) on Spain, Italy, Nigeria, and the Democratic Republic of the Congo (DRC) [4,38]. This validation process employed a non-linear least-squares method (NLSM) curve-fitting technique to estimate the optimal values of key unknown parameters. The model was calibrated using weekly confirmed cases from these countries, representing the number of infectious individuals. Prior to the model validation, a time-series analysis of the published data was conducted, highlighting the trends in weekly reported cases through a 7-day moving average, shown in Figure 2a. The visualized time series also captures the weekly progression of Mpox cases for each country, as shown in Figure 2b,c, and provides an overall comparative analysis, depicted in Figure 2. This approach helped to identify patterns and anomalies in the spread of the disease before the model was fitted to the observed data.

**Figure 3:**
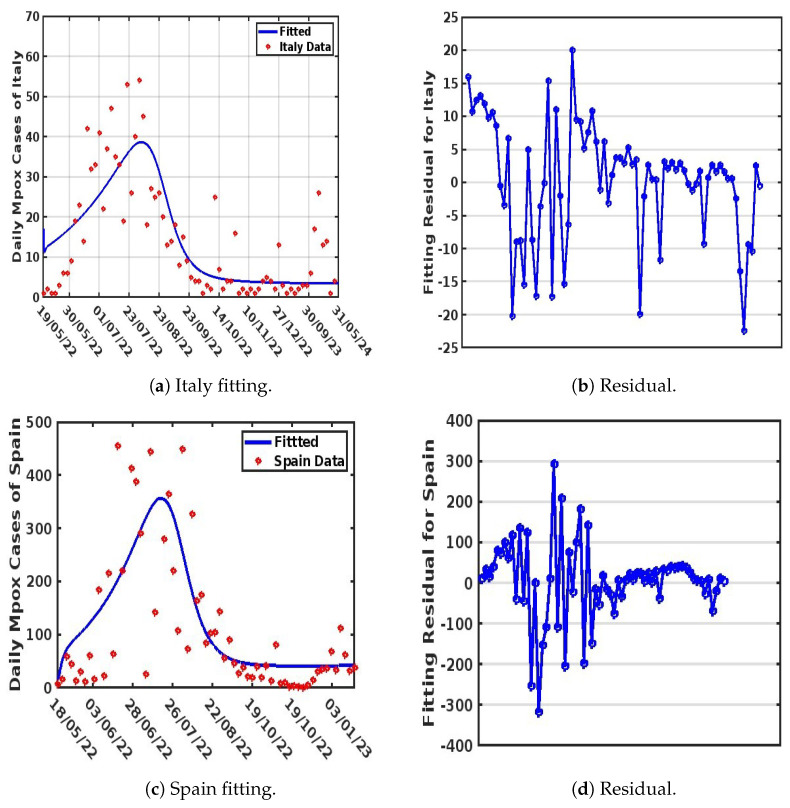
Model fitting for Italy and Spain.

**Figure 4:**
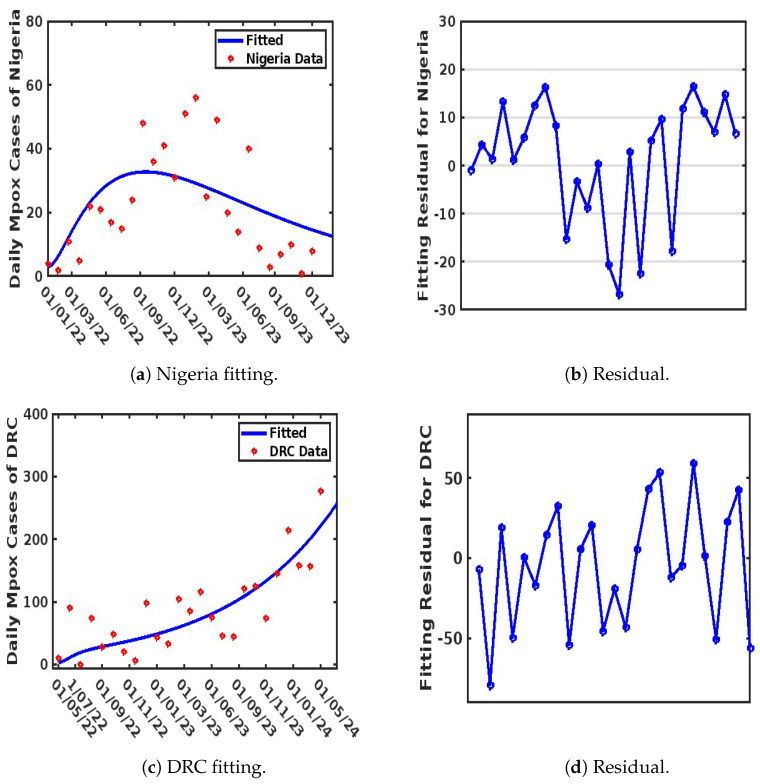
Model fitting for Nigeria and the DRC.

The weekly reported Mpox cases from the WHO [4] were used to drive the fitting process, which utilized MATLAB’s “lsqcurvefit” algorithm to minimize the sum of the squared differences between each data point obtained. This process was repeated 1000 times for each parameter. The goodness of fit was evaluated using the Root Mean Squared Error (RMSE), resulting in values of 8.9028, 97.9763, 12.3850, and 37.3720 for Italy, Spain, Nigeria, and the DRC, respectively. The obtained values for known parameters, along with unknown parameter values, are presented in Table 2 and Table 3 respectively, with model fits illustrated in Figure 3 and Figure 4.

It is important to note that parameter selection was guided by their significance in capturing the transmission dynamics of Mpox, particularly in a population with more than one strain of the virus. We used the residual function from the MATLAB Optimization Toolbox to generate the residual plots shown in Figure 3b,d and Figure 4b,d. These plots demonstrate a justified trend, with residuals evenly scattered above and below the y-origin. Residuals are essential for identifying outlying y-values and validating the linear regression assumptions concerning the error term in the regression model. High-leverage observations usually have smaller residuals as they tend to pull the regression line or surface closer to them, representing the vertical distance between the observed data and the fitted curve. Thus, when the residuals are randomly scattered around the y-origin, it indicates a justified fitting, as illustrated in Figure 3a,c and Figure 4a,c.

## 4. Statistical Data Modeling

In this section, we aim to fit the weekly reported Mpox cases data for Spain, Nigeria, Italy, and the DRC to two classical non-linear statistical models, which will serve as a foundation for the comparison of our mathematical model fit at the population level to the statistical fits.

### 4.1. Generalized Additive Modeling (GAM)

Linear models are easy to use, and the interpretation of their parameters is straightforward. However, when modeling complex non-linear phenomena, it is beneficial to use models that can represent non-linear relationships while maintaining good predictive performance. In such cases, generalized additive models (GAMs) provide a valuable alternative [40]. GAMs allow for flexibility by incorporating non-linear relationships between the response variable and the predictors while still being interpretable. A generalized additive model is expressed as the sum of smooth functions and is defined as follows:

(8) $$y_{i}=b_{0}+\sum_{p}s_{p}\left(x_{pi}\right)+\epsilon_{i},$$

where $y_{i}$ can belong to any exponential family distribution, which, in this context, represents the weekly reported cases of Mpox in Spain, Italy, Nigeria, and the Democratic Republic of Congo (DRC). The error term, $\epsilon_{i}$, is assumed to follow a normal distribution, $\epsilon_{i}$∼$N(0,\sigma^{2})$, with a mean of 0 and constant variance $\sigma^{2}$. The term $b_{0}$ represents the intercept and $s_{p}\left(x_{pi}\right)$ are smooth functions of the predictors $x_{pi}$, which, in this case, represent time measured in weeks. Using the GAM function in the R software [40], we represent the smooth function as the Gaussian process smoothed using 20 knots, and *ciTools* was used to estimate the confidence interval (CI).

The goodness of fit was evaluated using the Root Mean Squared Error (RMSE), resulting in values of 6.218943, 90.64003, 9.090687, and 19.84252 for Italy, Spain, Nigeria, and the DRC, respectively.

### 4.2. Generalized Linear Modeling (GLM)

Generalized linear models (GLMs) are statistical models that fit a linear response function $y_{i}$, which, in this context, represents the weekly reported cases of Mpox in Spain, Italy, Nigeria, and the Democratic Republic of Congo (DRC) as a linear combination of the predictors $x_{pi}$. In this case, the predictors represent time measured in weeks. The model accounts for errors $\epsilon_{i}$, which are assumed to be normally distributed [40]. A generalized linear model can be expressed as follows:

(9) $$y_{i}=b_{0}+\sum_{p}b_{p}x_{pi}+\epsilon_{i},$$

where $y_{i}$ belongs to any exponential family distribution, such as the Binomial, Bernoulli, Gamma, Normal, Gaussian, or Poisson distributions. In this context, we used the GLM function in the R software [40], representing $y_{i}$, a Gaussian distribution. The *ciTools* package [41] was employed to estimate the confidence interval (CI).

The error term, $\epsilon_{i}$, is assumed to follow a normal distribution, $\epsilon_{i}$∼$N(0,\sigma^{2})$, with a mean of 0 and constant variance $\sigma^{2}$. The intercept is denoted by $b_{0}$, and the coefficients $b_{p}$ are typically estimated using the ordinary least-squares method.

The goodness of fit was evaluated using the Root Mean Squared Error (RMSE), resulting in values of 10.79793, 136.9384, 16.80437, and 26.38759 for Italy, Spain, Nigeria, and the DRC, respectively.

### 4.3. Comparison of the Strain Dynamics Mathematical Model Developed with Classical Statistical Data Modeling at the Population Level

**Figure 5:**
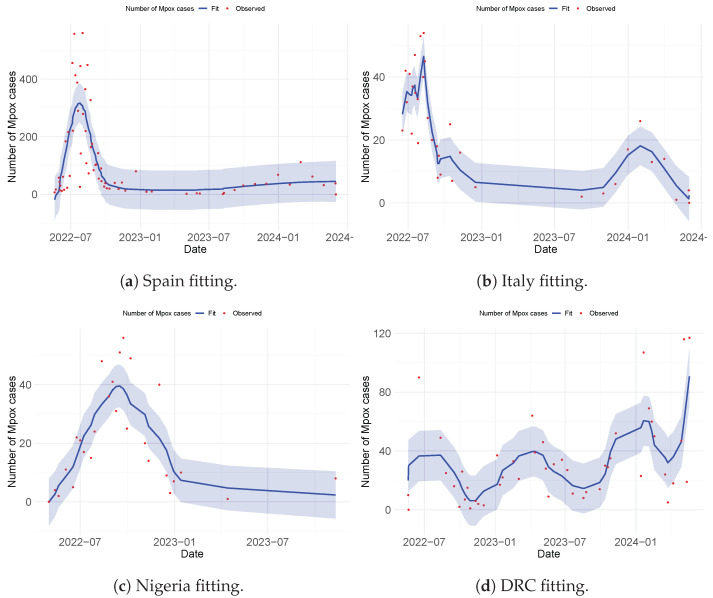
Fitting using the statistical model (GAM) for Spain, Italy, Nigeria, and the DRC. The error bar in blue represents the confidence interval (CI).

**Figure 6:**
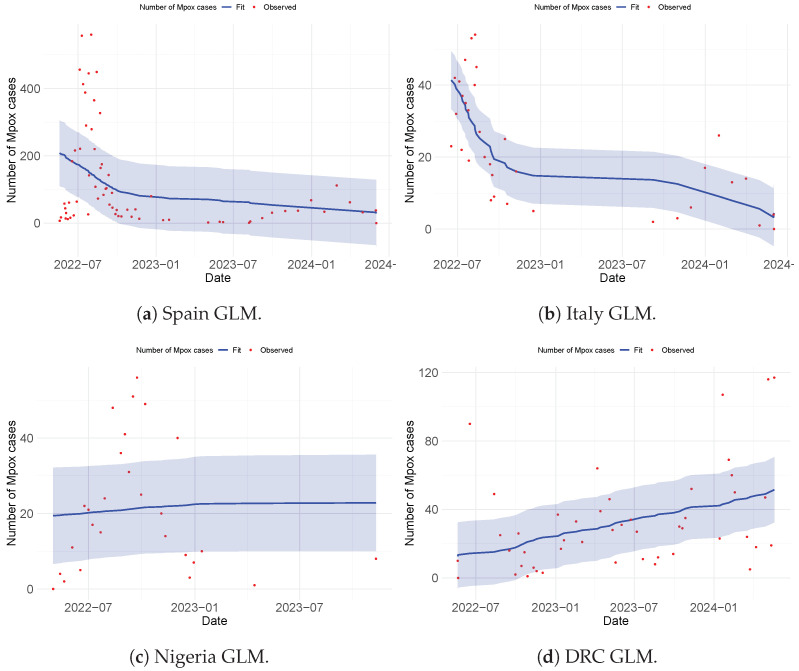
Fitting using the statistical model (GLM) for Spain, Italy, Nigeria, and the DRC. The error bar in blue represents the confidence interval (CI).

Here, we discuss the various approaches adopted to fit the observed Mpox data obtained from the WHO website [38]. This analysis was performed to guide the authors in selecting an appropriate approach for model fitting and parameter estimation to be employed in the formulated model. Three distinct methods were used to perform the data fitting and evaluate the Root Mean Squared Error (RMSE) to determine the best fit. These methods are the non-linear least-squares method (NLSM), generalized additive modeling (GAM), and generalized linear modeling (GLM). The observed data were applied to different models: Model (4) used the NLSM, Model (8) used GAM, and Model (9) used GLM. Among the three methods, GAM provided the best fit for the observed data (see Figure 5), achieving RMSE values of 6.218943 (Italy), 90.64003 (Spain), 9.090687 (Nigeria), and 19.84252 (DRC). The NLSM also performed well in fitting the observed data to Model (4) (see Figure 3 and Figure 4), with RMSE values of 8.9028 (Italy), 97.9763 (Spain), 12.3850 (Nigeria), and 37.3720 (DRC). In contrast, GLM provided the least accurate fit when applied to Model (9) (see Figure 6), with RMSE values of 10.79793 (Italy), 136.9384 (Spain), 16.80437 (Nigeria), and 26.38759 (DRC).

Importantly, while all three methods (NLSM, GAM, and GLM) were evaluated, the NLSM demonstrated a unique advantage. The NLSM not only provided competitive RMSE values but was also capable of estimating the parameter values for model (4), as presented in Table 3. This makes the NLSM a suitable choice for parameter estimation while maintaining a good balance of accuracy in terms of RMSE. In addition, while GAM and GLM could fit the data at the population level, the mathematical model developed was able to incorporate different complexities that are observed in Mpox spread dynamics and to estimate their parameters, which also allowed us to account for Mpox strain, which is the major focus of this work.

## 5. Mathematical Model Simulation for Different Scenarios

In this section, we illustrate the key mechanisms driving the dynamics of our model (4) through a series of graphical representations. These figures depict the system’s behavior in different scenarios, depending on whether the threshold parameter, $R_{0}$, is above or below unity. The figures are consistent with the analytical findings presented earlier. The parameter values used for the simulations are provided in Table 2 and Table 3, where $\Pi_{h}$ denotes the weekly recruitment rate into the population [42]. The initial conditions were based on the selected country’s (Nigeria, the DRC, Spain, and Italy) population, estimated at N(0)=(218,541,216,111,022,508,47,900,346,and59,342,867) [38]. Furthermore, the initial conditions for the state variables were determined in proportion to each country’s population. Specifically, the exposed population (*E*) was set to 0.225×N, the population infected with Clade I ($I_{1}$) to 0.003×N, the population infected with Clade II ($I_{2}$) to 0.015×N, the hospitalized population (*H*) to 0.075×N, and the recovered population (*R*) to 0.0076×N. The susceptible population (*S*) was then calculated as $S=N-E-I_{1}-I_{2}-H-R$. In order to thoroughly investigate the model dynamics (4), three distinct scenarios were considered, providing a comprehensive analysis of the system’s behavior:

i The effect of both Clades on the prevalence of Mpox within the population.
ii The influence of the effective contact rate on the prevalence of Mpox Clade I.
iii The impact of the effective contact rate on the prevalence of Mpox Clade II.

### 5.1. Analyze the Effect of Both Clades on the Prevalence of Mpox Within the Population

**Figure 7:**
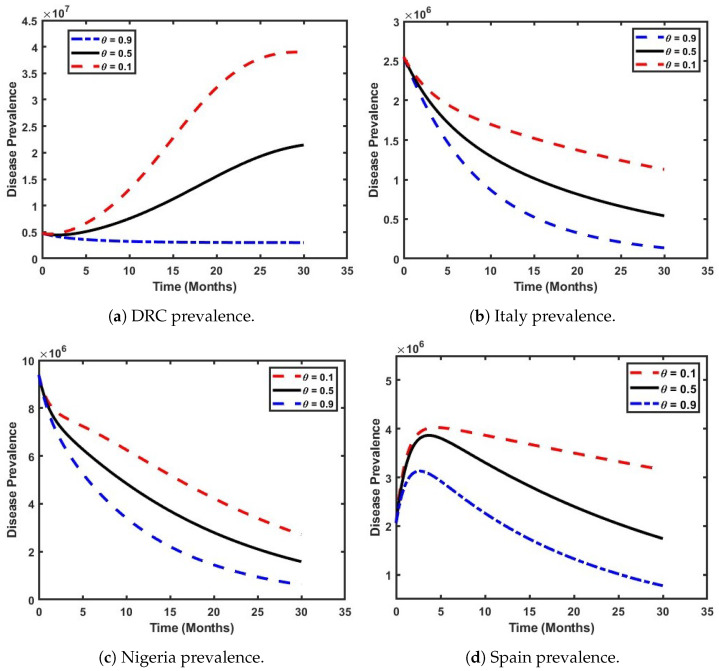
Impact of Clade I and Clade II Mpox prevalence across the countries.

In this section, we evaluate the effect of varying the parameter θ, which represents the proportion of individuals infected with either Clade I or Clade II of the Mpox disease. Figure 7 illustrates a scenario where both Clades coexist within the population. The figure reveals that the prevalence of Mpox in the DRC is strongly influenced by the dominant Clade. Notably, the prevalence is highest (indicated by the red dashed line) when 90% of the population is infected with Clade I. When the proportions of both Clades are equal (black solid line), the prevalence is higher than when Clade II constitutes only 10% of the infections (blue dash-dotted line). Additionally, as shown in Figure 7a, there is a steady increase in prevalence over time (red dashed line), rising from 0.5 million to 3.8 million individuals within 2.5 years. In Figure 7b, a similar pattern can be observed in the Italian population, where the prevalence of Mpox decreases as θ increases from 0.1 to 0.9. This corresponds to a decline in Italy’s basic reproduction number ($R_{0}$), with values of 0.7653, 0.4277, and 0.0900, respectively, within the same period. This indicates that the prevalence of Mpox in Italy is strongly influenced by the dominance of Clade I. Specifically, when 90% of the population is infected with Clade I (represented by the red dashed line), the prevalence is highest. Furthermore, it is observed that, when the proportions of both Clades are equal (represented by the black solid line), the prevalence remains higher compared to in the scenario where Clade II accounts for only 10% of the infections (represented by the blue dash-dotted line).

Furthermore, Figure 7c shows a significant decline in the prevalence of Mpox in the Nigerian population, regardless of the existing Mpox Clade. This suggests that Mpox can potentially be eradicated from the Nigerian populace, as an increase in θ leads to a corresponding decrease in the reproduction number ($R_{0}$), with values of 0.2724, 0.1519, and 0.0314, respectively, in the time period. Notably, the dominance of Mpox Clade II in the population is projected to reduce to its barest minimum within 2.5 years. Figure 7d illustrates a gradual increase in Mpox prevalence within the first three months of the outbreak in the Spanish population, regardless of the dominant Clade, but especially if it is Clade II. Following the fourth month, a steady decline in the number of infected individuals can be observed (which may be attributed to government interventions), as 2022 marked Spain’s first recorded Mpox outbreak in history. Notably, the predominance of the Clade II infection shows a higher Mpox prevalence compared to in the other selected countries (refer to the dark solid lines in the online version).

### 5.2. Examine How the Effective Contact Rate Influences the Prevalence of Mpox Clade I

**Figure 8:**
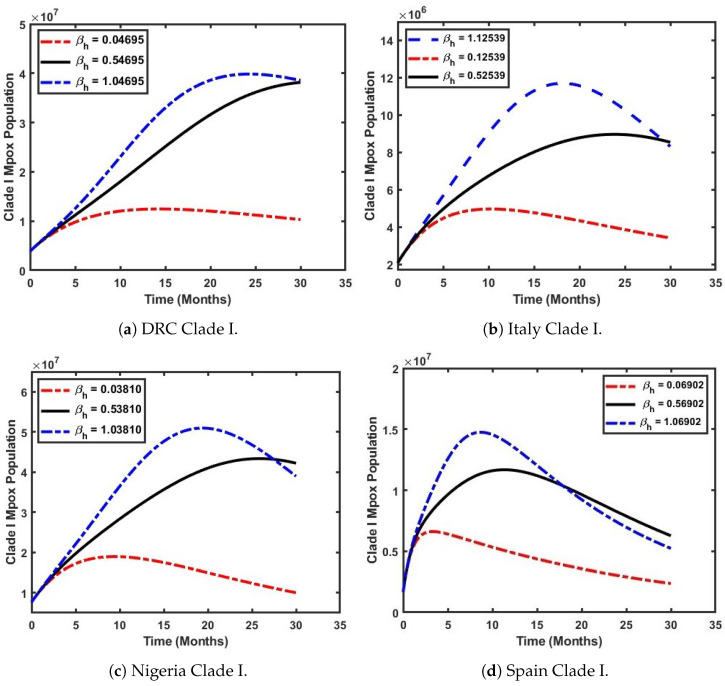
Varying the effective contact rate ($\beta_{h}$) to simulate the impact of Clade I Mpox across selected countries.

In Figure 8, we focus on the temporal variation of the effective contact rate ($\beta_{h}$) and its impact on Mpox Clade I. The parameters listed in Table 2 and Table 3 were used for the simulations, while ($\beta_{h}$) was varied, with a 50% (black solid line) increase and a 100% (blue dashed line) increase used to evaluate the disease’s impact on the population over time. Figure 8a illustrates the scenario in the DRC population when $\beta_{h}$, the effective contact rate, is varied from its baseline value (indicated by the red dashed line). It can be observed that an increase in $\beta_{h}$ significantly influences the basic reproduction number, $R_{0}$. When $\beta_{h}=0.04695$, $R_{0}=0.5995$, which is less than unity. At this level, the disease does not spread exponentially. However, when $\beta_{h}$ is increased by 50%, the population of the dominant Clade I grows exponentially from 0.5 million infected individuals to 3.8 million within 2.5 years (black solid line). In contrast, a 100% increase in $\beta_{h}$ results in $R_{0}=13.3678$. Under this condition, the population of infected individuals shows a steady increase within the first 15 months (1 year and 3 months) until it reaches a peak, maintaining this level for up to 2.5 years (blue dashed line). Also, a similar pattern can be observed in Figure 8b. However, for Italy, a 100% increase in the effective contact rate results in $R_{0}=7.6261$. In this scenario, the infected population experiences a sharp increase from 2 million to 11 million within the first 1.5 years of the outbreak, before gradually declining to 7 million over 2.5 years (blue dashed line). This indicates that a 100% increase in the effective contact rate in Italy would strongly amplify the spread of Clade I and should be avoided.

Furthermore, Figure 8c illustrates the impact of an increasing effective contact rate ($\beta_{h}$) on the population of Nigeria. It reveals that Mpox Clade I emerges as the dominant strain, as higher values of $\beta_{h}$ lead to an elevated basic reproduction number, resulting in a significant rise in the number of infected individuals. At $\beta_{h}=0.53810$, a consistent increase in the infected population (depicted by the black solid line) is observed, growing from 0.9 million to 4.2 million individuals over a period of 2.5 years. However, this trend highlights the critical need for the Nigerian government to implement proactive measures to control the rise in the effective contact rate and prevent the disease from escalating into a widespread epidemic should Clade I spread into the country, which is a situation that can occur due to migration within Africa. In Figure 8d, it is shown that an increase in $\beta_{h}$ has a limited impact on Mpox Clade I within the Spanish population. A 50% rise in the effective reproduction number results in an increase in infections caused by Clade I (depicted by the black solid line) from 0.3 million to 1.4 million, followed by a gradual decline to 0.7 million over a 2.5-year period. This trend indicates that Clade II is the dominant strain in Spain.

### 5.3. Assess the Impact of the Effective Contact Rate on the Prevalence of Mpox Clade II

**Figure 9:**
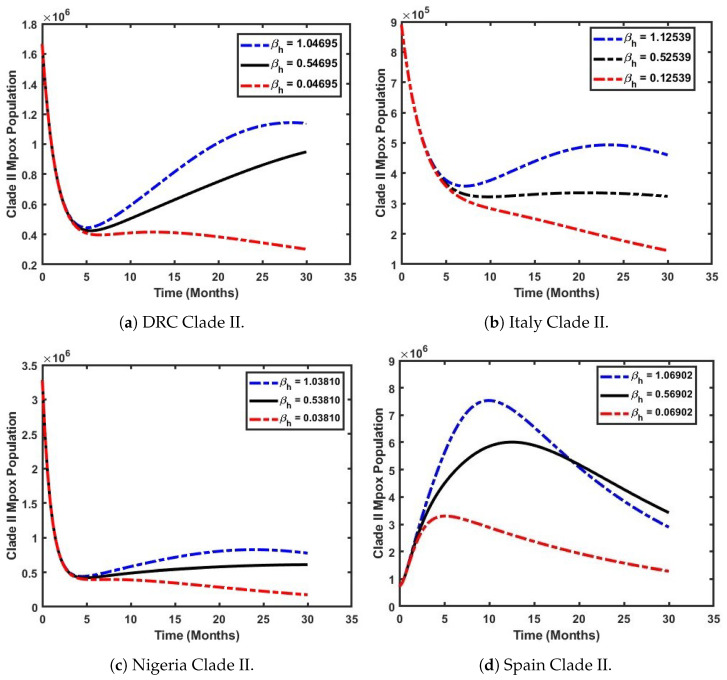
Varying the effective contact rate ($\beta_{h}$) to simulate the impact of Clade II Mpox across selected countries.

In this section, we assess the impact of the effective contact rate on the prevalence of Mpox Clade II using the formulated model (4) and the parameters presented in Table 2 and Table 3. In Figure 9, the effective contact rate is $\beta_{h}$. Figure 9a–c show a sharp decline in the dominance of Clade II in the populations of Italy, the DRC, and Nigeria within the first five years. Following this decline, a slight increase in the number of individuals infected with Clade II is observed. On the contrary, Figure 9d reveals a distinct trend. An increase in $\beta_{h}$ leads to a corresponding rise in the dominance of Clade II in Spain’s population. Specifically, when the effective contact rate is increased from its baseline value of 0.06902 to 1.06902 (a 100% increase), the number of infected individuals with Clade II surges dramatically. In Spain, infections rise from 0.8 million to 7.8 million within the first year (i.e., 12 months) of Clade II’s emergence, before declining to 3.4 million within 1.5 years. This insight highlights the need for immediate and robust mitigation strategies in Spain to prevent the widespread transmission of Mpox Clade II. The Spanish government should prioritize vaccination programs, early detection mechanisms, and effective treatment plans to manage the outbreak and minimize its impact on the population.

## 6. Concluding Remarks

### 6.1. Conclusion and Recommendation

This study investigated the epidemiological dynamics of Mpox across selected countries, utilizing a derived model to estimate the basic reproduction number ($R_{0}$) based on estimated parameters obtained from the observed Mpox data outlined in Table 3. The findings reveal that the persistence of Mpox in a population is strongly linked to the basic reproduction number exceeding unity, and the analysis reveals that higher values of $R_{0}$ correspond to sustained disease transmission and potential outbreaks. Further analysis was conducted to explore the impact of increased Mpox contact rates, simulating 50% and 100% rises in contact rates to better understand the disease dynamics in Spain, Nigeria, Italy, and the Democratic Republic of Congo (DRC). The results highlighted that, in Spain, the prevalence of Clade II Mpox could escalate dramatically, with the number of infected individuals potentially reaching 7 million under a 100% increase in contact rates. A similar trend was observed for Clade I Mpox in Italy (should Clade I spread into the country, which is a situation that can occur due to the influx of migrants into the country) and the DRC, indicating a significant risk of widespread transmission if contact rates continue to rise unchecked.

These findings emphasize the critical need for immediate and comprehensive mitigation strategies to curtail the spread of Mpox in Spain, Italy, and the DRC. Priority should be given to robust vaccination programs, early detection systems, public health education campaigns, and effective treatment protocols. Furthermore, governments and health organizations must invest in surveillance mechanisms to monitor changes in transmission dynamics and adapt their interventions accordingly. By implementing targeted and proactive measures, the risk of Mpox outbreaks can be minimized, thereby safeguarding public health and mitigating the socio-economic impacts of the disease.

### 6.2. Limitations and Future Work

The formulated model 4 has certain limitations that the authors wish to address in the future to enhance its applicability and realism in capturing the dynamics of Mpox transmission. Highlighting these limitations is critical for refining the model to produce more accurate and actionable insights, which can better inform policymakers and guide effective decision-making.

- The current formulation of the model (4) does not explicitly incorporate a vaccination compartment. Including a vaccination compartment in the model would significantly improve its ability to capture the effects of vaccination programs on the different Mpox strain dynamics. Vaccination plays a crucial role in mitigating the spread of Mpox, and its absence in the current formulation may lead to an incomplete representation of the real-world epidemiological scenario.
- The exposed compartment in Model (4) is not subdivided to account for the two distinct Mpox Clades: Clade I and Clade II. Subdividing the exposed compartment into these Clades is essential for capturing the heterogeneous nature of Mpox disease dynamics. Clade-specific characteristics, such as differences in transmissibility, severity, and geographic distribution, can have significant implications for understanding the spread of the disease and designing targeted interventions. Incorporating this distinction would make the model more biologically relevant and aligned with observed data.
- For future analyses, the authors propose the adoption of a time-dependent effective contact rate and control reproduction number as part of the modeling framework. Both parameters are critical metrics for assessing the effectiveness of interventions over time. By incorporating this dynamic measure, the model could provide a more detailed understanding of how control measures, such as vaccination and quarantine, impact the spread of the disease during the different stages of an outbreak.
- The authors recommend incorporating forecasting approaches into future analyses of the model. Forecasting techniques, such as machine learning or Bayesian inference, can enhance the predictive capabilities of the model by leveraging historical and real-time data. These methods can provide valuable insights into the potential trajectory of Mpox future outbreaks, allowing policymakers to make proactive and informed decisions regarding resource allocation, vaccination campaigns, and other interventions.
- It was observed that Clade II spread into different countries across the globe from its origin in West Africa due to immigration. We propose that incorporating movement into the model and extending it to a spatial model will account for the spatial spread of the disease, which will help to inform decisions at different spatial scales.
- Disparities in vaccination distribution and access to proper healthcare are still an issue in low-income countries where the disease is predominant; hence, investigating these health inequalities will enhance intervention strategies and the mitigation of Mpox spread in those countries.
- People infected with Mpox with underlying conditions (immunocompromised individuals) face increased risk and mortality from the disease, underscoring the interplay between co-morbid conditions and disease outcomes. Future models need to incorporate this factor into their formulation, perhaps by means of age structure modeling, which will take into account co-morbidity variables in children and young and older populations.

## Figures and Tables

**Table 1:** Description of variables and parameters used in Model (4).

Variable	Interpretation
*S*	Population of susceptible individuals
*E*	Population of individuals exposed to both Clades
$I_{1}$	Population of Clade I infectious individuals
$I_{2}$	Population of Clade II infectious individuals
*H*	Population of hospitalized individuals
*R*	Population of recovered individuals
$S_{m}$	Population of susceptible mammals
$I_{m}$	Population of infectious mammals
**Parameter**	**Interpretation**
$\Pi_{h}$	Recruitment rates into the human population
$\lambda_{h}$	Transmission rate of individuals to the exposed class from the susceptible class
ϵ	Re-infection rate or loss of immunity of recovered individuals for both Clades
γ	Transmission rate of individuals from exposed class to infectious compartment for Clade I and Clade II
θ	The proportion of individuals infected
$\tau_{1}$	Hospitalized rate of individuals in Clade I infectious population
$\tau_{2}$	Hospitalized rate of individuals in Clade II infectious population
$\delta_{1}$	Disease-induced death rate in Clade I infectious population
$\delta_{2}$	Disease-induced death rate in Clade II infectious population
$\delta_{3}$	Disease-induced death rate in infectious population in the hospitalized class
η	Recovery rate of individuals from hospitalized class
ψ	Recovery rate of individuals from Clade II infectious population
$\mu_{h}$	Natural mortality rate of human population
$\Pi_{m}$	Recruitment rates into the vector population
$\lambda_{m}$	Transmission rate of susceptible vector to infectious vector
μ	Natural mortality rate of the vector population
α	Modification parameters that reduce the infection transmission rate between humans and mammals

**Table 2:** Model (4) parameter values cited from the literature for Spain, Italy, Nigeria, and the Democratic Republic of the Congo. Some parameters $\left(\Pi_{m},\;\delta_{1},\;\delta_{1},\delta_{3}\right)$ were hypothetically chosen, while others were chosen from the literature ($\Pi_{h}$ [39], μ [1], γ [2,3,25]).

Country	$\Pi_{h}$	μ	γ	$\Pi_{m}$	$\delta_{1}$	$\delta_{2}$	$\delta_{3}$
Italy	687.30	0.0121	0.0714	1393.5	0.0180	0.1039	0.0004
Spain	2330.3	0.0118	0.0714	9559.5	0.0009	0.4551	0.0003
Nigeria	5329.8	0.0185	0.0714	1083.9	0.0320	0.5761	0.0076
DRC	8259.8	0.0163	0.0714	956.8	0.0001	0.5739	0.0102

**Table 3:** Estimated parameter values of Model (4) for the DRC, Nigeria, Italy, and Spain.

Parameter	Spain	Nigeria	DRC	Italy
ϵ	2.300 $\times \;10^{-4}$	8.800 $\times \;10^{-4}$	1.870 $\times \;10^{-1}$	4.3 $\times \;10^{-5}$
$\beta_{m}$	4.466 $\times \;10^{1}$	5.419 $\times \;10^{-1}$	6.728 $\times \;10^{1}$	2.5404 $\times \;10^{1}$
θ	5.000 $\times \;10^{-5}$	3.300 $\times \;10^{-4}$	2.000 $\times \;10^{-2}$	5.0 $\times \;10^{-4}$
$u_{m}$	4.10 $\times \;10^{-2}$	2.300 $\times \;10^{-2}$	2.151 $\times \;10^{-1}$	5.043 $\times \;10^{-1}$
ψ	3.090 $\times \;10^{-2}$	1.160 $\times \;10^{-2}$	5.300 $\times \;10^{-4}$	2.212 $\times \;10^{-1}$
η	0.789 $\times \;10^{1}$	1.038 $\times \;10^{-1}$	8.023 $\times \;10^{-1}$	1.3080 $\times \;10^{1}$
$\tau_{1}$	0.4645 $\times \;10^{1}$	0.4954 $\times \;10^{1}$	0.4610 $\times \;10^{1}$	0.96082 $\times \;10^{1}$
$\tau_{2}$	3.853 $\times \;10^{-1}$	3.922 $\times \;10^{-1}$	0.1553 $\times \;10^{1}$	0.27450 $\times \;10^{1}$
$\beta_{hm}$	0.2677 $\times \;10^{1}$	0.1810 $\times \;10^{1}$	0.2254 $\times \;10^{1}$	1.25396 $\times \;10^{1}$
σ	0.31741 $\times \;10^{1}$	0.4117 $\times \;10^{1}$	0.9819 $\times \;10^{1}$	0.18953 $\times \;10^{1}$
$\beta_{h}$	0.6902 $\times \;10^{1}$	0.3810 $\times \;10^{1}$	0.4695 $\times \;10^{1}$	1.25396 $\times \;10^{1}$
γ	0.1072 $\times \;10^{1}$	6.284 $\times \;10^{-1}$	8.703 $\times \;10^{-1}$	0.14317 $\times \;10^{1}$
α	0.2311 $\times \;10^{1}$	3.3360 $\times \;10^{-1}$	3.888 $\times \;10^{-1}$	0.75697 $\times \;10^{1}$

## Data Availability

All used data are publicly available, and the code used to generate the plots can be requested from the corresponding author.

## Appendix A. Mathematical Model Analysis

Here, we aim to qualitatively analyze the dynamic properties of the Mpox strain dynamics model (4).

### Appendix A.1. Positivity and Boundedness

For Model (4) to be epidemiologically meaningful, it is imperative to show that all its state variables are non-negative for all times (t) and that D is indeed bounded. Thus, we adopt the following theorem:

**Theorem** **A1.**
*Let the initial values for Model (4) be $S\left(0\right)\geq 0,\;E\left(0\right)\geq 0,\;I_{1}\left(0\right)\geq 0,\;I_{2}\left(0\right)\geq 0,\;H\left(0\right)\geq 0,\;R\left(0\right)\geq 0,\;S_{m}\left(0\right)\geq 0,\;l_{m}\left(0\right)\geq 0$. Then, the solutions $\left(S,\;E,\;I_{1},\;I_{2}\;H,\;R,\;S_{m}\;I_{m}\right)$ are positive for all times (t>0).*

**Proof.** Let $t_{1}=\sup t>0:S>0,\;E>0,\;I_{1}>0,\;I_{2}>0,\;H>0,\;R>0,\;S_{m}>0,\;I_{m}>0\in \left[0,\;t\right].$ Thus, $t_{1}>0$. We have, from the first equation of Model (4),

$$\frac{dS}{dt}=-\left(\lambda_{h}+\mu_{h}\right)S,\;where\;\lambda_{h}=\frac{\beta_{h}\left(I_{1}+\sigma I_{2}\right)}{N_{h}}$$

which can be written as follows:

$$\int \frac{dS}{dt}=-\int \frac{dS}{dt},$$

so that

$$S\left(t_{1}\right)=S\left(0\right)\exp\left[-\int_{0}^{t_{1}}\lambda (u).du\right]>0,$$

Similarly, it can be shown that $E>0,\;I_{1}>0,\;I_{2}>0\;,H>0,\;R>0,\;S_{m}>0,\;I_{m}>0$. □

The SEIHR-SI formulated model is represented by the differential equations in system (4), which is to be analyzed in a feasible region D, and all state variables and parameters of the model are assumed to be positive, ∀t≥0. The bounded region is obtained through the following lemma:

**Lemma** **A1.**
*The compact D, defined as ($\Delta_{1}\times \Delta_{2}$), is a positively invariant set which attracts all positive orbits in $R_{+}^{8}$.*

**Proof.** According to the system of Equation (4), we have the following: Proof: Since $N_{h}\left(t\right)=S\left(t\right)+E\left(t\right)+I_{1}\left(t\right)+I_{2}\left(t\right)+R_{h}\left(t\right)$ and $N_{m}\left(t\right)=S_{m}\left(t\right)+I_{m}\left(t\right)$, the derivative of $N_{h}\left(t\right)$ is given by

(A1) $$\frac{dN_{h}}{dt}=\frac{dS}{dt}+\frac{dE}{dt}+\frac{dI_{1}}{dt}+\frac{d1_{2}}{dt}+\frac{dH}{dt}+\frac{dR}{dt}$$

and

(A2) $$\frac{dN_{m}}{dt}=\frac{dS_{m}}{dt}+\frac{dI_{m}}{dt}$$

Simplifying Equation (A1) becomes

(A3) $$\begin{array}{c} \frac{dN_{h}}{dt}=\Pi_{h}-\mu_{h}N_{h}-(\delta_{1}I_{h}+\delta_{2}I_{2}+\delta_{3}H \end{array}$$

Since $\delta_{1},\;\delta_{2},\;\delta_{3}>0$ and $I_{1}\left(t\right)\;I_{2}\left(t\right),\;H\left(t\right),\geq 0$ for all t≥0, Equation (A3) becomes

(A4) $$\begin{array}{c} \frac{dN_{h}}{dt}\leq \Pi_{h}-\mu_{h}N_{h} \end{array}$$

Integrating Equation (A4) and solving gives

(A5) $$\begin{array}{cc} N_{h}\left(t\right)\leq & \;\frac{\Pi_{h}}{\mu_{h}}+\left(N_{h}\left(0\right)-\frac{\Pi_{h}}{\mu_{h}}\right)e^{-\mu_{h}t}\\ \leq & \;N_{h}\left(0\right)e^{-\mu_{h}t}+\frac{\Pi_{h}}{\mu_{h}}\left(1-e^{-\mu_{h}t}\right) \end{array}$$

Similarly, solving the second equation of (A2) gives

(A6) $$\begin{array}{cc} N_{m}\left(t\right)\leq & \;\frac{\Pi_{m}}{\phi +\delta }+\left(N_{R}\left(0\right)-\frac{\Pi_{m}}{\mu_{m}}\right)e^{-\mu_{m}t}\\ \leq & \;N_{m}\left(0\right)e^{-\mu_{m}t}+\frac{\Pi_{m}}{\mu_{m}}\left(1-e^{-\mu_{m}t}\right) \end{array}$$

Therefore, as $t\geq 0,\;N_{h}\left(t\right)\leq \frac{\Pi_{h}}{\mu_{h}}$ and $N_{m}\left(t\right)\leq \frac{\Pi_{m}}{\mu_{m}}$. Hence

(A7) $$\begin{array}{cc} \Omega_{1}= & \left\{\left(S,\;E,\;I_{1}\;I_{2}\;H,\;R_{h}\right)\in R_{+}^{6}:N_{h}\leq \frac{\Pi_{h}}{\mu_{h}}\right\}\\ \Omega_{2}= & \left\{\left(S_{m},\;I_{m}\right)\in R_{+}^{2}:N_{m}\leq \frac{\Pi_{m}}{\mu_{m}}\right\}\\ D= & \left\{\Omega_{1}\times \Omega_{2}|N_{h}\leq \frac{\Pi_{h}}{\mu_{h}},N_{m}\leq \frac{\Pi_{m}}{\mu_{m}}\right\} \end{array}$$

□

This implies that $\frac{\Pi_{h}}{\mu_{h}}$ and $\frac{\Pi_{m}}{\mu_{m}}$ are the upper bound for the human population $N_{h}\left(t\right)$ and mammal reservoirs $N_{m}\left(t\right)$, respectively. Thus, from Lemma A1, the set Ω is positive invariant with respect to system (4). Then, we conclude that the set Ω is positively invariant and all solutions of system (4) are non-negative and epidemiologically well posed.

### Appendix A.2. Disease-Free Equilibrium

At the disease-free equilibrium (DFE), the compartment $E=I_{1}=I_{2}=H=R=l_{m}=0.$ The DFE of the model $E_{0}=\left(S^{0},E^{0},I_{1}^{0},I_{2}^{0},H^{0},R^{0},S_{m}^{0},l_{m}^{0}\right)$ is given as

$$E_{0}=\left(\frac{\Pi_{h}}{\mu_{h}},0,0,0,0,0,\frac{\Pi_{m}}{\mu_{m}},0\right)$$

To compute the basic reproduction number $R_{0}$, we use the next-generation matrix approach. The rate of transfer vectors into and out of the affected compartments *f* and *v*, respectively, are given by

$$f=\left[\begin{array}{c} \frac{S\beta_{h}\left(I_{1}+I_{2}\sigma \right)}{N_{h}}\\ 0\\ 0\\ S_{m}\left(\frac{I_{m}\beta_{m}}{N_{m}}+\frac{\alpha \beta_{hm}\left(I_{1}+I_{2}\sigma \right)}{N_{h}}\right) \end{array}\right],\;v=\left[\begin{array}{c} E\left(\gamma_{h}+\mu_{h}\right)\\ -E\gamma_{h}\left(1-\theta \right)+I_{1}\left(\delta_{1}+\mu_{h}+\tau_{1}\right)\\ -E\gamma_{h}\theta +I_{2}\left(\delta_{2}+\mu_{h}+\psi +\tau_{2}\right)\\ H\left(\delta_{3}+\eta +\mu_{h}\right)-I_{1}\tau_{1}-I_{2}\tau_{2}\\ I_{m}\mu_{m} \end{array}\right]$$

The Jacobian matrix of *f* and *v* is given by

$$F=\left[\begin{array}{ccccc} 0 & \beta_{h} & \beta_{h}\sigma & 0 & 0\\ 0 & 0 & 0 & 0 & 0\\ 0 & 0 & 0 & 0 & 0\\ 0 & 0 & 0 & 0 & 0\\ 0 & \frac{\Pi_{m}\alpha \beta_{hm}\mu_{h}}{\Pi_{h}\mu_{m}} & \frac{\Pi_{m}\alpha \beta_{hm}\mu_{h}\sigma }{\Pi_{h}\mu_{m}} & 0 & \frac{\Pi_{m}\beta_{m}}{N_{m}\mu_{m}} \end{array}\right],\;\mathrm{and}\;V=\left[\begin{array}{ccccc} m_{0} & 0 & 0 & 0 & 0\\ -\gamma_{h}\left(1-\theta \right) & m_{1} & 0 & 0 & 0\\ -\gamma_{h}\theta & 0 & m_{3} & 0 & 0\\ 0 & -\tau_{1} & -\tau_{2} & m_{4} & 0\\ 0 & 0 & 0 & 0 & \mu_{m} \end{array}\right]$$

So that

(A8) $$\begin{array}{cc} \rho \left(FV^{-1}\right)=R_{0} & =\frac{\beta_{h}\gamma_{h}\left[\sigma \theta \left(\delta_{1}+\mu_{h}+\tau_{1}\right)+\left(1-\theta \right)\left(\delta_{2}+\mu_{h}+\psi +\tau_{2}\right)\right]}{\left(\gamma_{h}+\mu_{h}\right)\left(\delta_{1}+\mu_{h}+\tau_{1}\right)\left(\delta_{2}+\mu_{h}+\psi +\tau_{2}\right)} \end{array}$$

(A9) $$\begin{array}{cc} \; & =\frac{\beta_{h}\gamma_{h}\left[\sigma \theta m_{1}+\left(1-\theta \right)m_{2}\right]}{m_{0}m_{1}m_{2}} \end{array}$$

where $R_{0}$ is positive since all parameters are positive and 0≤θ≤1.

### Appendix A.3. Existence and Uniqueness of Solution

We show that Equation (4) has a unique solution $\left(S,\;E,\;I_{1},\;I_{2},\;H,\;R,\;S_{m},\;l_{m}\right)\in R_{+}^{8}$ under the condition of Equation (5) using the Lipschitz condition.

Consider a general form of ODE $y^{\prime }=f\left(x,y\right)$ with $y\left(x_{0}\right)=y_{0},x\in R$. If *f* is continuous and has partial derivatives that are continuous in the region R, then f(x,y) is Lipschitz continuous.

We re-write Model (4) in the form F(x) with solution $x\in R_{+}^{8}$:

(A10) $$\begin{array}{cc} F_{1} & =\Pi_{h}-\left(\lambda_{h}+\mu_{h}\right)S+\epsilon R,\\ F_{2} & =\lambda_{h}S-\gamma E_{h}-\mu_{h}E_{h}\\ F_{3} & =\left(1-\theta \right)\gamma E_{h}-\left(\tau_{1}+\delta_{1}+\mu_{h}\right)I_{1},\\ F_{4} & =\theta \gamma E_{h}-\left(\tau_{2}+\delta_{2}+\mu_{h}+\psi \right)I_{2},\\ F_{5} & =\tau_{1}I_{1}+\tau_{2}I_{2}-\left(\delta_{3}+\mu_{h}+\eta \right)H\\ F_{6} & =\eta H+\psi I_{2}-\left(\epsilon +\mu_{h}\right)R,\\ F_{7} & =\Pi_{m}-\lambda_{m}S_{m}-\mu_{m}S_{m},\\ F_{8} & =\lambda_{m}S_{m}-\mu_{m}I_{m}, \end{array}$$

**Lemma** **A2.**
*Let F(x) be continuous differentiable at $x\in R_{+}^{8}$.*

**Theorem** **A2.**
*Model (4) has a unique solution if F(x) is continuous and has a continuous partial derivative.*

**Proof.** Some partial derivatives of F(x) are given below.

$$\begin{array}{c} \frac{\partial F_{1}}{\partial x_{1}}=|-\left(\lambda_{h}+\mu_{h}\right)|<\infty \;\frac{\partial F_{1}}{\partial x_{2}}=0<\infty ;\;\frac{\partial F_{1}}{\partial x_{3}}=|\frac{\beta_{h}x_{1}}{N_{h}}|<\infty \;\frac{\partial F_{1}}{\partial x_{4}}=\left|\frac{\beta_{h}\sigma x_{1}}{N_{h}}\right|<\infty \\ \frac{\partial F_{1}}{\partial x_{5}}=0<\infty ;\;\frac{\partial F_{1}}{\partial x_{6}}=\epsilon <\infty \;\frac{\partial F_{1}}{\partial x_{7}}=0<\infty ;\;\frac{\partial F_{1}}{\partial x_{8}}=0<\infty \end{array}$$

The rest of the partial derivatives exist, are continuous, and are bounded in the same way as Equation (A10). Hence, according to Theorem A2, the model (4) has a unique solution. □

#### Endemic Equilibrium

Model (4) has an endemic equilibrium $E_{1}=\left(S^{**},E^{**},I_{1}^{**},I_{2}^{**},H^{**},R^{**},S_{m}^{**},l_{m}^{**}\right)$ with $E_{1}=0$, which can be given as $S_{m}^{**}=\frac{\Pi_{m}}{\lambda_{m}^{*}+\mu_{m}}$, $I_{m}^{**}=\frac{\Pi_{m}}{\mu_{m}\left(\lambda_{m}^{*}+\mu_{m}\right)}$, $E^{**}=\frac{\lambda_{h}^{**}S_{h}^{**}}{m_{0}}$,

$I_{1}^{**}=\frac{\left(1-\theta \right)\gamma \lambda_{h}^{**}S_{h}^{**}}{m_{0}m_{1}}$ $I_{2}^{**}=\frac{\theta \gamma \lambda_{h}^{**}S_{h}^{**}}{m_{0}m_{2}}$ $H^{**}=\frac{\tau_{1}\left(1-\theta \right)\gamma m_{2}\lambda_{h}^{**}S_{h}^{**}+\tau_{2}\theta \gamma m_{1}\lambda_{h}^{**}S_{h}^{**}}{m_{0}m_{1}m_{2}m_{3}},R^{**}=\frac{\eta \tau_{1}\left(1-\theta \right)\gamma m_{2}\lambda_{h}^{**}S_{h}^{**}+\eta \tau_{2}\theta \gamma m_{1}\lambda_{h}^{**}S_{h}^{**}+\psi \theta m_{1}m_{2}\gamma \lambda_{h}^{**}S^{**}}{m_{0}m_{1}m_{2}m_{3}m_{4}}$

Substituting Equation into the forces of infection $\lambda_{h}^{**}$ and $\lambda_{m}^{**}$ and simplifying such that

$$\lambda_{h}^{**}=\frac{\beta_{h}\left(I_{1}^{**}+\sigma I_{2}^{**}\right)}{N_{h}},\lambda_{m}=\frac{\alpha \beta_{hm}\left(I_{1}^{**}+\sigma I_{2}^{**}\right)}{N_{h}}+\frac{\beta_{m}I_{m}^{**}}{N_{m}}$$

we have

$$p_{1}{\left(\lambda_{h}^{**}\right)}^{2}+p_{2}\left(\lambda_{h}^{**}\right)=0$$

where $m_{0}=\left(\gamma +\mu_{h}\right)$, $m_{1}=\left(\tau_{1}+\delta_{1}+\mu_{h}\right)$, $m_{2}=\left(\tau_{2}+\delta_{2}++\mu_{h}+\psi \right)$, $m_{3}=\delta_{3}+\mu_{h}+\eta$, $m_{4}=\left(\epsilon +\mu_{h}\right)$, $p_{1}=m_{1}m_{2}m_{3}m_{4}\Pi_{h}+m_{2}m_{3}m_{4}\left(1-\theta \right)\gamma \Pi_{h}+m_{1}m_{3}m_{4}\theta \gamma \Pi_{h}+m_{0}m_{4}\tau_{1}\left(1-\theta \right)\gamma \Pi_{h}+m_{1}m_{4}\tau_{2}\theta \gamma \Pi_{h}+\eta m_{0}\tau_{1}\left(1-\theta \right)\gamma \Pi_{h}+\eta m_{1}\tau_{2}\theta \gamma \Pi_{h}$, $p_{2}=M_{0}m_{1}m_{2}m_{3}m_{4}\Pi_{h}-\beta_{h}m_{2}m_{3}m_{4}\left(1-\theta \right)\gamma \Pi_{h}-\beta_{h}m_{1}m_{3}m_{4}\sigma \theta \gamma \Pi_{h}$.

### Appendix A.4. Local Stability of Mpox-Free Equilibrium

We analyze the local stability of the Mpox-free equilibrium of the model system (4) by using the control reproduction number $R_{0}$ in the following theorem, as described in [42]:

**Theorem** **A3.**
*The Mpox-free equilibrium $E_{0}$ of model (4) is locally asymptotically stable in the biological feasible region D if $R_{0}<1$ and unstable if $R_{0}>1$.*

**Proof.** In order to prove Theorem A3, the Jacobian matrix of model (4) is obtained at the Mpox disease-free equilibrium $E_{0}$, where $E=I_{1}=I_{2}=H=R=I_{m}=0$, and $N_{h}=S$ and $N_{m}=S_{m}$, as

(A11) $$J_{\left(E_{0}\right)}=\left[\begin{array}{cccccccc} -\mu_{h} & 0 & -\beta_{h} & -\sigma \beta_{h} & 0 & \epsilon & 0 & 0\\ \;\\ 0 & -(\gamma +\mu_{h}) & \beta_{h} & \sigma \beta_{h} & 0 & 0 & 0 & 0\\ \;\\ 0 & (1-\theta )\gamma & -(\tau_{1}+\delta_{1}+\mu_{h}) & 0 & 0 & 0 & 0 & 0\\ \;\\ 0 & \theta \gamma & 0 & -(\tau_{2}+\delta_{2}+\mu_{h}+\psi ) & 0 & 0 & 0 & 0\\ \;\\ 0 & 0 & \tau_{1} & \tau_{2} & -(\delta_{3}+\mu_{h}+\eta ) & 0 & 0 & 0\\ \;\\ 0 & 0 & 0 & \psi & \eta & -(\epsilon +\mu_{h}) & 0 & 0\\ \;\\ 0 & 0 & -\frac{\alpha \beta_{h}m_{m}\mu_{h}}{\mu_{m}\Pi_{h}} & -\frac{\sigma \alpha \beta_{h}m_{m}\mu_{h}}{\mu_{m}\Pi_{h}} & 0 & 0 & -\mu_{m} & -\beta_{m}\\ \;\\ 0 & 0 & \frac{\alpha \beta_{h}m_{m}\mu_{h}}{\mu_{m}\Pi_{h}} & \frac{\sigma \alpha \beta_{h}m_{m}\mu_{h}}{\mu_{m}\Pi_{h}} & 0 & 0 & 0 & -(\mu_{m}-\beta_{m})\\ \; \end{array}\right]$$

According to Equation (A11), it is sufficient to show that all the eigenvalues of $E_{0}$ are negative. We obtain the first four eigenvalues, $-\mu_{h},\;-\mu_{m}\;-\left(\epsilon +\mu_{h}\right),\;-\left(\delta_{3}+\eta +\mu_{h}\right),\;-\left(\mu_{m}-\beta_{m}\right)$, while the remaining eigenvalues can be obtained from the sub-matrix $J_{1}\left(E_{0}\right)$, which is given by

(A12) $${J_{1}}_{\left(E_{0}\right)}=\left[\begin{array}{ccc} -(\gamma +\mu_{h}) & \beta_{h} & \sigma \beta_{h}\\ (1-\theta )\gamma & -(\tau_{1}+\delta_{1}+\mu_{h}) & 0\\ \theta \gamma & 0 & -(\tau_{2}+\delta_{2}+\mu_{h}+\psi ) \end{array}\right]=\left[\begin{array}{ccc} -m_{0} & \beta_{h} & \sigma \beta_{h}\\ (1-\theta )\gamma & -m_{1} & 0\\ \theta \gamma & 0 & -m_{2} \end{array}\right]$$

The solutions of the characteristic polynomial for Equation (A12) are given as

(A13) $$x^{3}+\phi_{1}x^{2}+\phi_{2}x+\phi_{3}=0$$

where

$\phi_{1}=m_{2}+m_{1}+m_{0}$;
$\phi_{2}=m_{0}\left(m_{1}+m_{2}\right)+m_{1}m_{2}-\beta_{h}\gamma \left[\left(1-\theta \right)+\theta \sigma \right]$;
$\phi_{3}=m_{1}m_{0}m_{2}\left(1-R_{0}\right)$.

Applying the Routh–Hurwitz criterion, the cubic Equation (A13) will have roots with negative real parts if and only if $\phi_{1}>0,\;\psi_{3}>0$ and $\phi_{1}\phi_{2}>\phi_{3}.$ Clearly, $\phi_{1}>0$ and $\phi_{3}>0,\;\left(if\;R_{0}<\infty \right)$. As a result, the disease-free equilibrium $\left(E_{0}\right)$ is locally asymptotically stable if $R_{0}<\infty$. □
